# Whole-Genome Sequencing and Annotation of a Drug-Resistant Extrapulmonary Clinical Isolate of Beijing Genotype Mycobacterium tuberculosis from Pune, India

**DOI:** 10.1128/genomeA.00504-18

**Published:** 2018-06-21

**Authors:** Swarupa M. Hatolkar, Rabindra N. Misra, Rajesh Mahato, Savita Jadhav

**Affiliations:** a Department of Microbiology, Dr. D. Y. Patil Medical College, Hospital & Research Centre, Dr. D. Y. Patil Vidyapeeth (DPU), Pune, India; b ArraryGen Technologies Pvt. Ltd., Pune, India

## Abstract

Whole-genome sequencing has emerged as a powerful tool to map genetic diversity among Mycobacterium tuberculosis isolates and identify the genomic signatures associated with drug resistance, pathogenesis, and disease transmission. Isolate LJ319 of the Mycobacterium tuberculosis complex (MTC)-Beijing genotype circulating in Maharashtra, India, which was obtained from the cerebrospinal fluid (CSF) of an immunocompetent patient, was subjected to whole-genome sequencing.

## GENOME ANNOUNCEMENT

Tuberculosis (TB) remains one of the leading causes of morbidity and mortality worldwide. Extrapulmonary tuberculosis (EPTB) constitutes around 15 to 20% of TB cases in immunocompetent individuals (1). Whole-genome sequencing has emerged as a powerful tool to map genetic diversity among Mycobacterium tuberculosis isolates and identify the genomic signatures associated with drug resistance, pathogenesis, and disease transmission. Several pulmonary isolates of M. tuberculosis have been sequenced over the years. However, availability of whole-genome sequences of M. tuberculosis isolates from extrapulmonary sites is limited. Whole-genome sequencing in conjunction with comprehensive drug susceptibility testing can reveal clinically relevant mutations associated with drug resistance (2, 3).

In the present research, Mycobacterium tuberculosis LJ319 of the M. tuberculosis complex (MTC)-Beijing genotype circulating in the population of the Maharashtra state, which was isolated from cerebrospinal fluid (CSF) from an immunocompetent patient, was subjected to whole-genome sequencing (4). Isolation was done on conventional Lowenstein Jensen (LJ) solid culture. The isolate was found to be resistant to rifampin and isoniazid, and multidrug resistance (MDR) was confirmed with a line probe assay and Sanger sequencing methods (5, 6).

The paired-end sequencing was performed on an Illumina MiSeq platform. High-quality reads were mapped to the genome of reference strain *M. tuberculosis* H37Rv (GenBank accession no. NC_000962) using SPAdes v. 3.11, generating a reference assembly with an average read-mapping coverage of 100×.

The National Center for Biotechnology Information (NCBI) Prokaryotic Genome Annotation Pipeline was used for the annotation of the reference assembly.

The total numbers of genes and coding sequences (CDSs) identiﬁed are 4,285 and 4,074, respectively. Three types of rRNA, namely, 5S, 16S, and 23S, have been annotated. There are 44 tRNAs and 71 genes that exhibited frameshift mutations.

We have performed spoligotyping of the same strain and after analysis found that it is an East Asian Beijing genotype with the 43-spacer binary code (0000000000000000000000000000000000111111111).

### Accession number(s).

The annotated whole-genome sequence has been deposited in the NCBI GenBank database with the accession no. CP026742.
